# A Non-Surgical Treatise on Diseases of the Prostate Gland and Adnexa

**Published:** 1905-12

**Authors:** 


A NON-SURGICAL TREATISE ON DISEASES OF THE PROSTATE GLAND AND ADNEXA. By George Whitfield Overall, A.B., M.D., Chicago. Rowe Publishing Co., Chicago.

A better name for the above book would be "The Treatment of Diseases of the Prostate Gland and Adnexa by Electrolysis and

Cataphoresis," as these methods are extensively recommended in the treatment of all the conditions mentioned. Much valuable information is contained in this manual, and Dr. Overall seems to have a clear idea of the relative importance of lesions of the urethra, prostate and seminal vesicles in causing symptoms referable to the genitourinary organs. In his treatment, however, overmuch is given of the electrical methods, and in the management of chronic prostatis the value of massage is underestimated. Apparently cataphoresis as described and massage ought to give excellent results in treating this disease. Dr. Overall is unnecessarily bitter in his criticism of the present methods of dealing with prostatic hypertrophy, as the cases he reports do not seem to have furnished unusually brilliant results. We believe he has also overestimated the value of electrolysis in the treatment of urethral strictures.

With the above exceptions we commend Dr. Overall's treatise to those interested in electrical treatment, and believe many good points may be derived from its perusal.

